# Subtle Differences in Physician Communication and Substantial Impacts on Patient Decision‐Making About Low‐Value Care: An Experimental Vignette Study

**DOI:** 10.1111/jep.70208

**Published:** 2025-07-09

**Authors:** Ji Sun Park, Ji‐Su Lee, Young Kyung Do

**Affiliations:** ^1^ Department of Health Policy and Management Seoul National University College of Medicine Jongno‐gu South Korea; ^2^ Graduate School of Data Science Korea Advanced Institute of Science and Technology Daejeon South Korea; ^3^ Institute of Health Policy and Management Seoul National University Medical Research Center Jongno‐gu South Korea

**Keywords:** health services research, patient‐centred care, value

## Abstract

**Rational, Aims and Objectives:**

While low‐value care may have multiple contributing factors, it ultimately arises within patient‐physician interactions, often influenced by communication. This study examines whether and the extent to which physician communication factors influence patients' willingness to undergo low‐value care using hypothetical vignettes in South Korea.

**Methods:**

We utilised data from a total of 1229 South Korean adults that included hypothetical vignettes on low‐value care. Participants were presented with vignettes involving minor head injury and the potential use of computed tomography (CT). We assessed changes in the proportion of individuals willing to undergo low‐value CT based on three physician communication factors: explicit recommendation, communication regarding test benefits and risks, and expressions indicative of defensive medicine, such as warning about missed problems and stating no legal responsibility. We also examined participant characteristics associated with their willingness to use low‐value CT.

**Results:**

Decisions regarding low‐value care were significantly influenced by physician communication factors. For instance, while 68.5% of participants were willing to use low‐value CT without explicit recommendation, 76.6% were willing when an explicit recommendation was made. Among those initially willing to use low‐value CT without an explicit recommendation, 54.6% changed their decision and chose not to undergo the CT when presented with information on the risks and benefits. However, 69% of these individuals reversed their decision and opted for the CT when the vignette included elements indicative of defensive medicine. The willingness to use low‐value care was not associated with most participant characteristics, except for supplementary private health insurance and individual preferences for health care.

**Conclusions:**

Physician communication plays a pivotal role in patient decision‐making regarding low‐value care. To reduce low‐value care, improving physician communication should be prioritised and considered a crucial leverage point in quality improvement initiatives.

## 1 Introduction

Low‐value care refers to medical services where the potential harm or costs outweigh the benefits for patients [1]. Low‐value care increases health care costs and can lead to direct physical harm, cascading tests, and the anxiety induced by false‐positive results [2]. Several factors contribute to its use, including the practice environment, the cultures of professional medicine and health care consumption, and individual patient and physician factors, all interacting within the health care system [3]. However, previous studies have primarily focused on investigating observable, system‐level factors such as fee‐for‐service payment structure, with less attention given to the subtler, deeply ingrained routine clinical practices and habits of clinicians and staff [2, 4].

Although low‐value care results from a complex interplay of system‐ and individual‐level factors, it fundamentally arises from patient‐physician interactions at the point of care [3]. This intricate dynamic extends beyond visible factors, encompassing nuanced and less quantifiable aspects of routine clinical practices. Understanding these subtleties is crucial, as seemingly minor differences in physician communication—whether in what is said or left unsaid—can significantly influence a patient's decision to opt for low‐value care [5]. Physicians may inadvertently nudge patients toward such care through slight changes in wording [6]. Furthermore, physician communication may fail to clearly convey essential information about the benefits and risks, increasing the likelihood of patients choosing low‐value care [3, 7]. Even when physicians explain that a particular service may have limited value, expressions rooted in defensive medicine such as stating that the physician is not liable for any problem missed due to not performing a test can still increase patient anxiety and lead to unnecessary testing [8]. Despite the recognised importance of physician communication, there has been limited empirical research investigating how subtle, often unintentional changes in communication that better reflect the complex interactions in real clinical settings influence or steer patient decisions regarding low‐value care. These nuanced aspects of communication remain insufficiently explored, leaving significant gaps in understanding both their impact and the extent to which such subtle differences influence patient decision‐making.

This study gap is partly due to the inherent challenges of observing or measuring physician‐patient encounters. Prior studies investigating factors influencing low‐value care often rely on administrative data, which lack detailed information about clinical interactions and face methodological challenges related to bias and the operational definition of low‐value care. To address these limitations, a promising alternative is the use of hypothetical vignettes. This method allows for the creation of scenarios involving low‐value care and enables the experimental testing of causal hypotheses, specifically focusing on how physician communication influences patient preferences for low‐value care while minimising confounding factors that are difficult to control in real‐life data [9].

The purpose of this study is to assess the influence of physician communication factors on patients' willingness to undergo potential low‐value computed tomography (CT) using hypothetical vignettes in South Korea. Additionally, we explore participant characteristics associated with preferences for low‐value CT.

## 2 Methods

### 2.1 Study Setting and Participants

**Figure 1:**
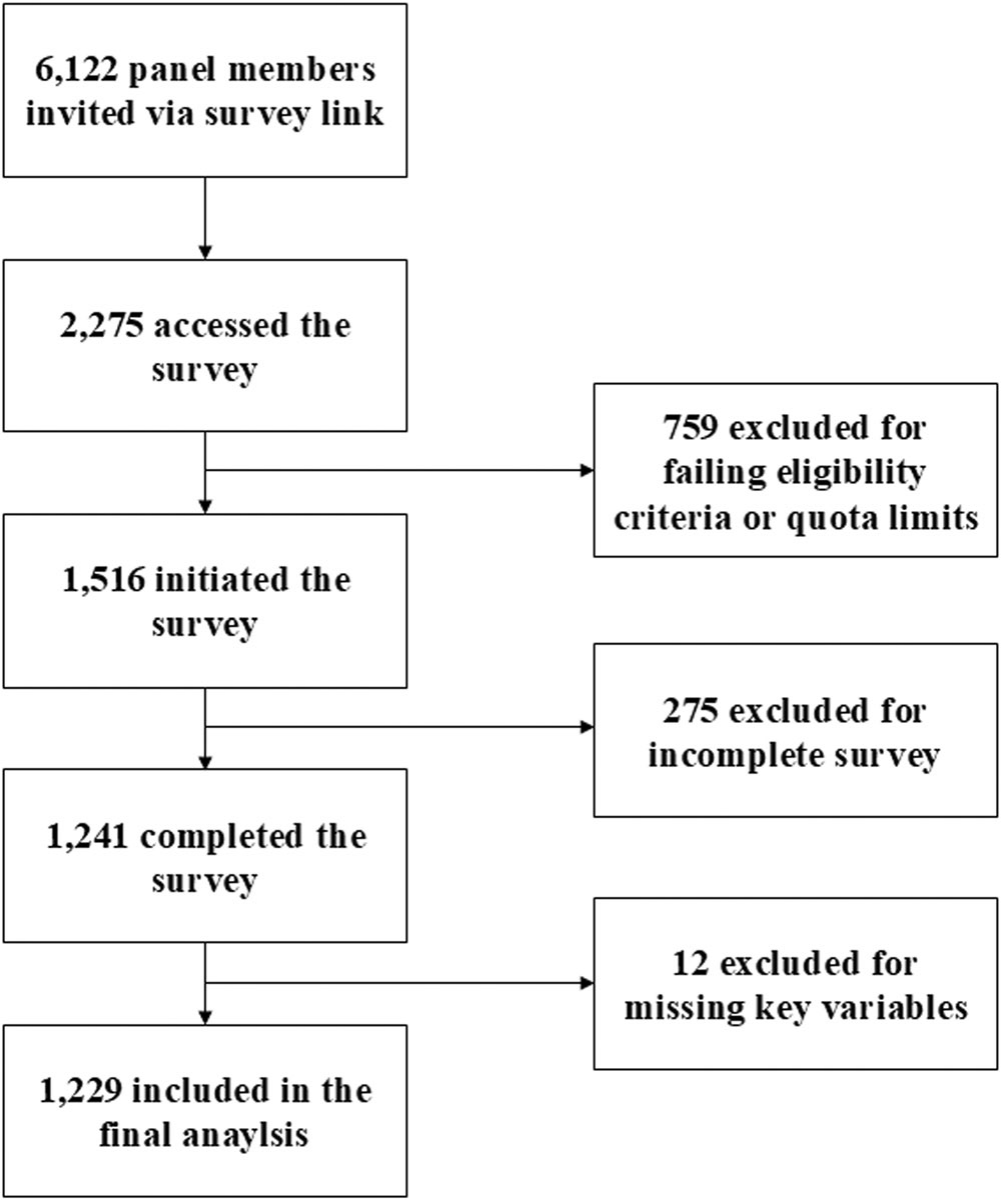
Flow of survey participants.

We employed a vignette‐based survey research design for this study, conducted online in South Korea between 24 December 2020 and 5 January 2021. The target population consisted of a nationally representative sample of South Korean adults aged 19−69, excluding individuals aged 70 and older due to lower internet literacy and potential difficulties in completing an online survey independently. Participants were recruited by Gallup Korea, a specialised market research company, using proportional quota sampling based on gender, age, and residential area. The survey link was sent to 6122 panel members of Gallup Korea, and 2275 individuals accessed the survey. Of these, 759 did not meet eligibility criteria, leaving 1516 individuals who started the survey. Among these, 1241 completed the full survey. Twelve responses had missing data on key variables, resulting in a final analytical sample of 1229 participants. The detailed participant flow is presented in Figure 1.

### 2.2 Vignettes and Design

We developed clinical vignettes that presented scenarios where participants were asked to consider low‐value care based on physician communication factors. These vignettes were informed by related literature [10, 11] and our clinical experience in the emergency department (ED). CT scans for low‐impact head injury in ED settings were selected as a case of low‐value care based on international clinical guidelines and recommendations. According to the American College of Emergency Physicians' top‐five list of low‐value emergency care practices and the Choosing Wisely campaign, head CTs are not recommended for patients with minor head injury who are at low risk for intracranial bleeding or skull fracture as determined by clinical decision rules [11, 12].

However, despite these recommendations, CT scans are used extensively in South Korea, where CT scans per 1000 population reached 281.5 in 2021—the highest rate among OECD member countries [13]. Although over 97% of South Koreans are covered by National Health Insurance (NHI), many enroll in private health insurance (PHI) to cover medical expenses not covered by NHI, including out‐of‐pocket costs for advanced imaging such as CT scans [14]. In South Korea, CT scans are often considered examples of low‐value care due to their cost, associated radiation risks, and the potential to trigger additional unnecessary tests or treatments.

Participants were presented with a clinical scenario in which they arrived at the ED following a minor head injury, with normal physical examination findings and symptoms. The scenario represented a low‐risk injury where CT scan utilisation was not clinically indicated and was considered most likely to be low‐value care. Based on the literature, three physician communication factors were identified as drivers of lower value‐care in frontline patient‐physician interactions: physician recommendation, poor communication regarding the benefits and risks of medical interventions, and defensive medicine [3, 6, 8]. These factors have been identified as major culprits in physician communication that can lead to deviations from optimal medical decision‐making [15]. Participants were asked whether they would be willing to undergo a CT scan when presented with a series of scenarios involving these physician communication factors, which influenced their decisions regarding low‐value care.

**Figure 2:**
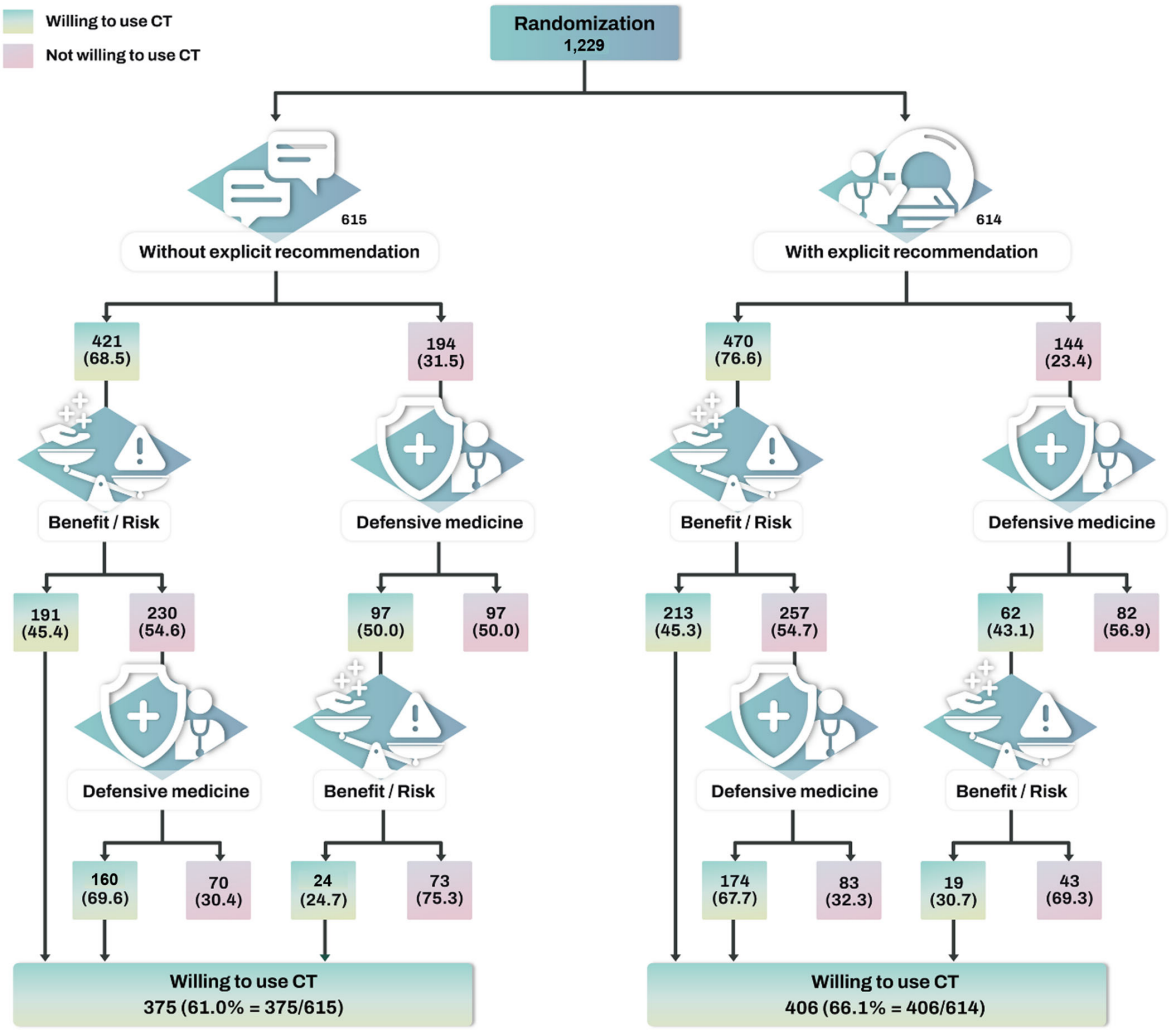
Proportion of participants willing to use low‐value CT across different scenarios. Frequency and conditional probabilities (in parentheses) presented. Participants were randomly assigned, with half receiving a vignette in which the physician told them they could undergo a brain CT scan for a more precise confirmation of the absence of any problems (‘Without explicit recommendation’), and the other half receiving a vignette in which the physician told them a brain CT scan is recommended (‘With explicit recommendation’). In the ‘Benefit/Risk’ scenario, participants were asked if they would use CT after the physician explained that a brain CT exposes the head to radiation and marginally increases the risk of brain tumours, with minimal benefits. Regarding ‘Defensive medicine’, participants were asked if they would use CT after the physician explained they would not bear liability for any missed problems due to not taking a CT. The complete vignettes scenarios can be found in online Supporting Information.

Participants were initially randomly assigned to one of two vignettes: in the first, the physician informed them that they could undergo a brain CT scan for more precise confirmation of the absence of any problems, while in the second, the physician explicitly recommended a brain CT scan. (The complete vignettes are available in the online Supporting Information S1: File S1). The vignette where the physician did not explicitly recommend the scan was referred to as *without explicit recommendation*, and the vignette where the physician recommended the scan was labelled as *with explicit recommendation*. Participants were then asked if they would be willing to use a brain CT in these situations. Those who were willing were subsequently presented with another vignette where the physician explained that a brain CT involves radiation exposure, marginally increases the risk of brain tumours, and offers minimal benefits. This vignette was termed as *provision of test benefits and risks information*, and participants were asked if they would still opt for the CT. Conversely, those who were initially unwilling to use the CT were asked again if they would opt for the CT after hearing that the physician would not bear liability for any missed problems due to foregoing the CT. This vignette was referred to as *defensive medicine*. The vignettes of *provision of test benefits and risks information* and *defensive medicine* were presented selectively in response to the participant's decision (See Figure 2 for the flow of vignettes presented).

### 2.3 Statistical Analysis

Descriptive statistics (counts and proportions) were used to summarise participants' willingness to undergo low‐value CT across the different scenarios. We conducted chi‐square tests to examine whether the differences in willingness to use CT between the two randomised groups (with vs. without explicit physician recommendation) were statistically significant at two points: the initial decision and the final decision after all subsequent information had been presented. Decision shifts at each subsequent information stage (i.e., benefit/risk information, defensive medicine information, and their sequences) were assessed for statistical significance using Fisher's exact test. Additional chi‐square tests were performed to examine potentially heterogenous decision shifts at each subsequent information stage between the two randomised groups. To identify participant characteristics associated with their eventual willingness, logistic regression analysis was performed. The participant characteristics included gender, age, marital status, education level, average monthly household income, health care coverage, self‐rated health status, comorbidity status, supplementary PHI, and Medical Maximiser‐Minimiser Scale (MMS) score. Chronic disease status was assessed via a single self‐reported item asking, ‘Do you have any long‐standing illness, chronic condition, or disability?’ The MMS is a validated 10‐item instrument used to assess individuals' general preference for medical intervention [16]. Responses are recorded on a 7‐point Likert scale (strongly disagree to strongly agree), with total scores ranging from 10 (lowest possible) to 70 (highest possible) and higher scores indicating a preference for more health care. All statistical analyses were conducted using Stata version 17.0 (StataCorp., College Station, TX, USA).

## 3 Results

The participants' characteristics are described in Table 1. With a mean age of 45 years, the majority of participants were married (60.8%), had a college‐level education or above (70.5%), had NHI coverage (96.6%), and had additional PHI (76.8%). Additionally, 65.6% reported being in good health, and 66.9% reported not having a chronic disease. The mean MMS score was 50 (Table 2).

**Table 1:** Characteristics of the participants.

	Full sample (*N* = 1229)	
Characteristics	*n* (%) or mean (SD)	
Gender		
Men	622	(50.6)
Women	607	(49.4)
Age	45	(13.5)
Marital status		
Not married	428	(34.8)
Married or cohabiting	747	(60.8)
Separated, divorced or widowed	54	(4.4)
Education level		
High school	249	(20.3)
Junior college	114	(9.3)
College or above	866	(70.5)
Average monthly household income (10,000 Korean won)		
(0, 200)	150	(12.2)
(200, 400)	340	(2.7)
(400, 600)	327	(26.6)
(600, 800)	149	(12.1)
(800, ~)	263	(21.4)
Health care coverage		
National health insurance	1,187	(96.6)
Medical aid	42	(3.4)
Self‐rated health status		
Healthy	806	(65.6)
Unhealthy or fair	423	(34.4)
Comorbidity status		
Without chronic disease	822	(66.9)
With chronic disease	407	(33.1)
Private health insurance		
No	285	(23.2)
Yes	944	(76.8)
Medical maximiser‐minimiser scale score	50	(7.9)

*Note:* Observations in the full sample (*N* = 1229) were used. Frequency and column percentage (in parentheses) are presented for categorical variables, and means and standard deviations (in parentheses) are presented for continuous variables.

Abbreviation: SD, standard deviations.

**Table 2:** Willingness to use low‐value CT by randomised group.

	Without explicit recommendation (A, %)	With explicit recommendation (B, %)	Difference in proportion (B–A, %*p*)	*p*‐value
Initial decision	68.5	76.6	+8.1	0.001
Final decision	61.0	66.1	+5.1	0.061

*Note:* The full sample was used. Chi‐square tests were conducted to examine whether willingness to undergo a low‐value CT scan was significantly different between the groups with and without explicit physician recommendation at the initial and final decision points.

The characteristics of the two groups that were randomly assigned to different physician communication scenarios were similar. A detailed comparison of these characteristics is provided in online Supporting Information S1: File S2.

The participants' decisions regarding the use of low‐value CT are presented in Figure 2. Without explicit recommendation from the physician, 68.5% of participants were initially willing to use low‐value CT, which is 8.1 percentage points lower than the 76.6% willingness observed in the group presented with explicit recommendation. Among those who initially expressed willingness to use low‐value CT without explicit recommendation, more than half (54.6%) changed their decision and opted not to undergo the CT scan after being presented with information on the benefits and risks. Among these individuals, 69.6% reversed their decision and chose to use CT when the defensive medicine vignette was presented. Even among those initially unwilling to use CT in the group without explicit recommendation, 50.0% changed their decision and chose to use CT after the defensive medicine vignette was presented. Among these individuals, 75.3% changed their decisions and opted not to use CT again after receiving information about the benefits and risks.

In the group with explicit recommendation, the proportion of choosing to undergo the CT scan was higher than in the group without explicit recommendation (76.6% vs. 68.5%). However, once within the explicit recommendation group, the magnitudes of decision shifts due to the presentation of benefit‐risk information or defensive medicine practice were largely similar to those shown in the group without explicit recommendation. For instance, 54.7% of those initially willing reversed their decision and chose not to use the CT scan after being presented with information about the benefits and risks. Among these individuals, 67.7% changed their decision again and chose to undergo the CT scan after the defensive medicine vignette was presented.

The eventual proportion of those willing to undergo the scan was 61.0% in the group without explicit recommendation and 66.1% in the group with explicit recommendation. This 5.1 percentage point difference was not statistically significant (*p* = 0.061) and somewhat attenuated compared to the initial difference of 8.1 percentage points, which was statistically significant (*p* = 0.010).

Decision shifts at each information stage are presented and compared between the two groups in Table 3. All decision shifts were substantial and statistically different from zero (*p* < 0.001). For example, as many as 75.3% of individuals who were initially unwilling to use CT without explicit recommendation but became willing when presented with the defensive medicine vignette, subsequently switched back to being unwilling when benefit/risk information was provided. The impact of physician communication on decision shifts did not differ between the two groups (all *p* > 0.05).

**Table 3:** Impact of physician communication on decision shifts regarding low‐value CT use, conditional on prior willingness or unwillingness.

	Without explicit recommendation (A, %)	With explicit recommendation (B, %)	Difference in proportion (B–A, %*p*)	*p* value^a^
*Prior willingness → Unwillingness*				
[1] Benefit/Risk	54.6	54.7	0.1	0.988
[2] Benefit/Risk following defensive medicine	75.3	69.3	–6.0	0.414
*Prior unwillingness → Willingness*				
[3] Defensive medicine	50.0	43.1	–6.9	0.206
[4] Defensive medicine following benefit/risk	69.6	67.7	–1.9	0.659

*Note:* [1] Communication of benefit/risk information conditional on prior willingness to undergo a CT scan either with or without explicit recommendation (randomisation); [2] Communication of benefit/risk information conditional on prior willingness to undergo a CT scan following defensive medicine; [3] Communication of defensive medicine conditional on prior unwillingness to undergo a CT scan either with or without explicit recommendation (randomisation); [4] Communication of defensive medicine on prior unwillingness to undergo a CT scan following benefit/risk. See Figure 2 for an illustration of the conditional nature of subsequent information provision. Fisher's exact tests were used to assess the statistical significance of individual decision shifts (all *p* < 0.001).

^a^ Chi‐square tests were conducted to examine whether decision shifts differed significantly between the two groups at each information stage.

Logistic regression results examining factors associated with the eventual willingness to use for low‐value CT are presented in Table 4. Participants assigned to the group with explicit recommendation were statistically significantly more likely to prefer low‐value CT compared to those without explicit recommendation, even after controlling for sociodemographic and health‐related factors (OR: 1.35, 95% CI: 1.03 to 1.06, *p* < 0.05). Participants with PHI were significantly more likely to desire low‐value CT when compared to those without PHI (OR: 1.68, 95% CI: 1.27 to 2.24, *p* < 0.001). Additionally, participants with higher MMS scores were more likely to report willingness to use low‐value CT (OR: 1.35, 95% CI: 1.03 to 1.06, *p* < 0.001). Participants' willingness to use low‐value CT was not statistically significantly associated with other sociodemographic and health related factors.

**Table 4:** Logistic regression of eventual willingness to use for low‐value CT.

Characteristics	OR	95% CI
Gender		
Men	Ref	
Women	0.92	0.72 to 1.17
Age	0.99	0.98 to 1.00
Marital status		
Not married	Ref	
Married or cohabiting	1.39	0.99 to 1.96
Separated, divorced or widowed	1.41	0.72 to 2.73
Education level		
Junior college	1.29	0.80 to 2.09
College or above	1.35	0.99 to 1.84
Log of average monthly household income	0.97	0.86 to 1.09
Health care coverage		
National health insurance	Ref	
Medical aid	0.90	0.47 to 1.74
Self‐rated health status		
Healthy	Ref	
Unhealthy or fair	1.02	0.78 to 1.33
Comorbidity status		
Without chronic disease	Ref	
With chronic disease	0.92	0.70 to 1.20
Private health insurance		
No	Ref	
Yes	1.68***	1.27 to 2.24
Medical maximiser‐minimiser scale	1.05***	1.03 to 1.06
Physician recommendation		
Without explicit recommendation	Ref	
With explicit recommendation	1.35*	1.03 to 1.06

*Note:* The full sample was used. Logistic regression model was used to examine whether using a low‐value CT was associated with characteristics of participants, and odds ratios (ORs) and 95% confidence intervals (CIs) were estimated. Participants' characteristics included in the model included gender, age, marital status, educational level, log of average monthly household income, health care coverage, self‐rated health status, comorbidity status, private health insurance, medical maximiser‐minimiser scale.

Abbreviations: CI, confidence interval; OR, odds ratio; Ref, reference category.

* *p* < 0.05;

*** *p* < 0.001.

## 4 Discussion

### 4.1 Discussion

The main findings of this vignette study indicate that physician communication substantially influences participants' preferences regarding low‐value CT scans. Our research demonstrates that even subtle changes in the wording of a physician's recommendation can affect the willingness to accept low‐value care. Additionally, participants' acceptance for low‐value care was swayed by communication that shifted decision‐making responsibility to the patient, often to mitigate the physician's medico‐legal risk. Importantly, providing information about the benefits/risks of the test discouraged more than half of the individuals at various stages of the clinical vignettes from opting for a low‐value CT scan. Notably, explicit physician recommendations initially increased willingness, but subsequent information, such as benefit/risk and defensive medicine explanations, seemed to moderate this effect. Furthermore, we found that the inclination toward low‐value care was generally not associated with most common individual characteristics, except for PHI and preferences for medical care.

Our results suggest that various aspects of physicians' communication exert considerable influence on patients' decision to choose low‐value care. The finding that physician recommendations can increase the utilisation of low‐value care aligns with previous studies [6, 17]. Multiple distal factors may influence physicians to recommend such care. For instance, physicians may induce demand for certain procedures, leveraging their knowledge that patients may lack, even when these procedures offer little or no benefit to the patient and may primarily serve the physician's interests [18]. Payment models and financial incentives significantly influence physicians' recommendations, contributing to the overuse of low‐value care. Beyond physician‐induced demand and financial incentives, other contributing factors include practice habits, training or education, and a prevailing culture that values ‘more is better’ [19].

Furthermore, our results emphasise the substantial impact of physician communication regarding treatment benefits and risks on low‐value care, supporting earlier studies [20, 21]. Patients tend to overestimate the benefits and underestimate the harms associated with medical care [7]. Thus, balanced communication that provides treatment advantages and disadvantages is essential for improving patients' expectations and discouraging their inclination toward low‐value care.

Moreover, our findings indicate that defensive medicine constitutes a critical barrier to reducing low‐value care, consistent with existing research [22, 23]. Defensive medicine can erode physician‐patient trust by framing patients as potential litigants rather than partners in care. When patients perceive that their physician is acting out of self‐protection rather than prioritising their well‐being, they may feel anxiety about forgoing a test or procedure, leading them to seek additional testing for reassurance, even when it offers little clinical benefit [24].

Our study also demonstrates that low‐value care is not particularly associated with most common patient characteristics, corroborating prior research that indicates no consistent patterns between patient characteristics and the use of low‐value care [25]. However, our results reveal that having supplementary PHI and greater preferences for health care use were significantly linked to low‐value care. This is consistent with existing literature [26, 27]. The relationship between medical maximiser‐minimiser preferences and the receipt of low‐value care has also been documented [28]. Medical maximisers, who tend to desire ‘everything done’, may partially explain the higher likelihood of patients receiving low‐value care, even when such interventions are unnecessary [28].

This study has two major strengths. First, it explored poorly understood aspects of physician communication during patient‐physician interactions using the vignette method. This method allowed us to examine whether and to what extent patients were influenced by subtle nuances in physicians' language and the information provided and not provided. Second, the study adopted a holistic perspective on physician communication factors, including explicit recommendations, benefits and risks information, and defensive medicine practices. These factors are interdependent and often inseparable in reality, highlighting the importance of an integrated understanding, as emphasised in recent literature [29].

Several limitations should be acknowledged. First, patient decision‐making in hypothetical vignettes may not accurately reflect real‐world decisions, raising concerns about the validity of our results [30, 31]. However, existing evidence suggests that responses in vignettes closely mirror those in real‐life situations [30]. Second, this study assessed participants' stated preferences for undergoing a low‐value CT scan rather than their actual utilisation of low‐value CT. However, as patient preferences often predict actual health care utilisation, further research using real‐world data is needed to confirm this relationship. Third, patients may interpret the physician communication factors specified in the vignette differently from our intention, making our results sensitive to the specific wording of the scenarios. Fourth, while we made deliberate efforts to reflect the nuanced aspects of physician communication, in reality, such communication is even more intricate than the scenarios we presented. Subtle differences in language, tone, and context—elements we sought to incorporate—may still be more varied in actual clinical settings. Furthermore, non‐verbal cues such as facial expressions and body language, which play a significant role in patient decision‐making, were not included in this study. Future research should further expand on these subtleties, exploring both verbal and non‐verbal communication styles to better represent the full complexity of physician‐patient interactions. Fifth, although the sample was constructed using stratified quota sampling based on sex, age, and region to ensure national representativeness, no adjustments were made for potential bias due to nonresponse or missing data. However, the proportion of such cases was relatively small, suggesting that the impact on the study findings is likely limited.

### 4.2 Conclusion

This study emphasises the substantial influence that subtle nuances and often inadvertent shifts in physician communication can have on patient decision‐making regarding low‐value care. By recognising how even small differences in phrasing and the presentation of information can steer patient perceptions and choices, this study underscores the critical role communication plays in such decisions. To improve physician communication and effectively reduce low‐value care, a multi‐pronged intervention may be necessary. This could involve cultivating a culture that discourages physicians from recommending care of little or no value, promoting the adoption of shared decision‐making (SDM), and fostering an environment that mitigates defensive medicine practices.

### 4.3 Practice Implications

While policies aimed at reducing low‐value care have predominantly concentrated on top‐down reforms such as payment reforms [2], our study suggests that bottom‐up interventions focusing on physicians' communication factors are equally important to decrease low‐value care. These communication factors play a vital role in shaping patient decisions and preferences, making them essential to address alongside existing policy initiatives. A crucial policy‐relevant issue is fostering a clinical and medico‐legal environment that promotes effective, patient‐centred physician communication. This involves providing clear information about the benefits and risks of care while minimising unnecessary patient anxiety and protecting physicians from unreasonable litigation risks. Without addressing physician communication directed at their patients, the impact of policies to reduce low‐value care would be limited, as such policies ultimately have to work through frontline patient‐physician interactions. Emphasising communication training and support for physicians—especially by addressing time constraints and physical strain due to exhaustion—can enhance patient understanding and decision‐making, thus improving the overall effectiveness of low‐value care reduction strategies.

To improve physician communication and effectively reduce low‐value care during clinical encounters, there is a pressing need for SDM [3]. SDM, as a model of good communication, has been shown to be effective in decreasing the prevalence of low‐value care [32]. Decision aids can be used to facilitate SDM and enhance communication about the benefits and risks of care [33, 34]. Genuinely engaging in SDM may also decrease a clinician's defensive attitude, as it promotes a collaborative effort between physicians and patients to make decisions together, sharing responsibility [35]. The Choosing Wisely initiative, which provides evidence‐based information for the public about interventions that are commonly used yet may be unnecessary or cause harm, may also help ensure patients have balanced information while serving as a reliable reference point for physicians [33]. More research is also needed to better understand physician communication behaviours that influence patient decisions regarding low‐value care.

## Ethics Statement

This study was approved by the Seoul National University Hospital Biomedical Research Institute (IRB Number: C‐2011‐190‐1178).

## Conflicts of Interest

The authors declare no conflicts of interest.

## Supporting information

Supplementary file1 submitted.

Supplementary file2 submitted.

## Data Availability

The data for this study were collected for a contract research supported by the Health Insurance Review and Assessment Service. Queries for data availability should be made to the corresponding author and the Health Insurance Review and Assessment Service.
