# Supplementary material for: Subtle Differences in Physician Communication and Substantial Impacts on Patient Decision‐Making About Low‐Value Care: An Experimental Vignette Study
Source: J Eval Clin Pract. 2025 Jul 9;31(5):e70208. doi: 10.1111/jep.70208 (PMC12239706; doi:10.1111/jep.70208)
Supplement: Supplementary file 1 — Supplementary file1 submitted. [file JEP-31-0-s001.docx]

**[Group 1: Without Explicit Recommendation]**

1. Let us consider the following scenario: This morning, you experienced a fall on the street, resulting in a head impact with the ground. Consequently, you developed pain and a bruise on your head, but did not exhibit symptoms such as blurred consciousness, loss of sensation in extremities, or impaired mobility. Upon visiting the emergency room for examination, you are told by the physician that there seemed to be no indication of skull fracture, brain hemorrhage, or brain swelling but that if you would like a more precise confirmation of the absence of any problems, you could undergo a brain CT scan, which would incur an approximate cost of 150,000 Korean Won. In this situation, would you choose to undergo the CT scan?

1. Yes (Go to 2.) ② No (Go to 4.)

2. Upon deciding to undergo the CT scan, you are told by the physician that undergoing a brain CT scan involves exposing the head to radiation and marginally increases the risk of brain tumors. Simultaneously, the physician adds that, given the current condition, the benefits derived from obtaining a CT scan are minimal. In this situation, would you choose to undergo the CT scan?

1. Yes (Exit of Module) ② No (Go to 3.)

3. Upon deciding not to undergo the CT scan, you are told by the physician that if you do not undergo the CT scan, it is possible to miss existing problems even though the probability may be very low. The physician adds that, because the information about the CT procedure has been provided to you, the medical team does not bear liability for having missed something as a result of not taking the CT scan. In this situation, would you choose to undergo the CT scan?

1. Yes (Exit of Module) ② No (Exit of Module)

4. Upon deciding not to undergo the CT scan, you are told by the physician that if you do not undergo the CT scan, it is possible to miss existing problems even though the probability may be very low. The physician adds that, because the information about the CT procedure has been provided to you, the medical team does not bear liability for having missed something as a result of not taking the CT scan. In this situation, would you choose to undergo the CT scan?

1. Yes (Go to 5.) ② No (Exit of Module)

5. Upon deciding to undergo the CT scan, you are told by the physician that undergoing a brain CT scan involves exposing the head to radiation and marginally increases the risk of brain tumors. Simultaneously, the physician adds that, given the current condition, the benefits derived from obtaining a CT scan are minimal. In this situation, would you choose to undergo the CT scan?

1. Yes (Exit of Module) ② No (Exit of Module)

**[Group 2: With Explicit Recommendation]**

1. Let us consider the following scenario: This morning, you experienced a fall on the street, resulting in a head impact with the ground. Consequently, you developed pain and a bruise on your head, but did not exhibit symptoms such as blurred consciousness, loss of sensation in extremities, or impaired mobility. Upon visiting the emergency room for examination, you are told by the physician that there seemed to be no indication of skull fracture, brain hemorrhage, or brain swelling but that for a more precise confirmation of the absence of any problems, a brain CT scan is recommended, which would incur an approximate cost of 150,000 Korean Won. In this situation, would you choose to undergo the CT scan?

1. Yes (Go to 2.) ② No (Go to 4.)

2. Upon deciding to undergo the CT scan, you are told by the physician that undergoing a brain CT scan involves exposing the head to radiation and marginally increases the risk of brain tumors. Simultaneously, the physician adds that, given the current condition, the benefits derived from obtaining a CT scan are minimal. In this situation, would you choose to undergo the CT scan?

1. Yes (Exit of Module) ② No (Go to 3.)

3. Upon deciding not to undergo the CT scan, you are told by the physician that if you do not undergo the CT scan, it is possible to miss existing problems even though the probability may be very low. The physician adds that, because the information about the CT procedure has been provided to you, the medical team does not bear liability for having missed something as a result of not taking the CT scan. In this situation, would you choose to undergo the CT scan?

1. Yes (Exit of Module) ② No (Exit of Module)

4. Upon deciding not to undergo the CT scan, you are told by the physician that if you do not undergo the CT scan, it is possible to miss existing problems even though the probability may be very low. The physician adds that, because the information about the CT procedure has been provided to you, the medical team does not bear liability for having missed something as a result of not taking the CT scan. In this situation, would you choose to undergo the CT scan?

1. Yes (Go to 5.) ② No (Exit of Module)

5. Upon deciding to undergo the CT scan, you are told by the physician that undergoing a brain CT scan involves exposing the head to radiation and marginally increases the risk of brain tumors. Simultaneously, the physician adds that, given the current condition, the benefits derived from obtaining a CT scan are minimal. In this situation, would you choose to undergo the CT scan?

1. Yes (Exit of Module) ② No (Exit of Module)
