# Supplementary material for: Subtle Differences in Physician Communication and Substantial Impacts on Patient Decision‐Making About Low‐Value Care: An Experimental Vignette Study
Source: J Eval Clin Pract. 2025 Jul 9;31(5):e70208. doi: 10.1111/jep.70208 (PMC12239706; doi:10.1111/jep.70208)
Supplement: Supplementary file 2 — Supplementary file2 submitted. [file JEP-31-0-s002.docx]

| **Characteristics** | Without explicit recommendation  (N=615)  n (%) or mean (SD) | | With explicit recommendation  (N=614)  n (%) or mean (SD) | |
| --- | --- | --- | --- | --- |
| **Gender** |  |  |  | |
| Men | 316 | (51.4) | 306 | (49.8) |
| Women | 299 | (48.6) | 308 | (50.2) |
| **Age** | 44 | (13.1) | 46 | (13.8) |
| **Marital status** |  |  |  |  |
| Not married | 220 | (35.8) | 208 | (33.9) |
| Married or cohabiting | 368 | (59.8) | 379 | (61.7) |
| Separated, divorced, or widowed | 27 | (4.4) | 27 | (4.4) |
| **Education level** |  |  |  |  |
| High school | 104 | (16.9) | 145 | (23.6) |
| Junior college | 57 | (9.3) | 57 | (9.3) |
| College or above | 454 | (73.8) | 412 | (67.1) |
| **Average monthly household income (10,000 Korean won)** |  |  |  |  |
| (0, 200] | 73 | (11.9) | 77 | (12.5) |
| (200, 400] | 180 | (29.3) | 160 | (26.1) |
| (400, 600] | 178 | (28.8) | 149 | (24.3) |
| (600, 800] | 65 | (10.6) | 84 | (13.7) |
| (800, ~] | 119 | (19.4) | 144 | (23.5) |
| **Healthcare coverage** |  |  |  |  |
| National Health Insurance | 594 | (96.6) | 593 | (96.6) |
| Medical Aid | 21 | (3.4) | 21 | (3.4) |
| **Self-rated health status** |  |  |  |  |
| Healthy | 404 | (65.7) | 402 | (65.5) |
| Unhealthy or fair | 211 | (34.3) | 212 | (34.5) |
| **Comorbidity status** |  |  |  |  |
| Without chronic disease | 420 | (68.3) | 402 | (65.5) |
| With chronic disease | 195 | (31.7) | 212 | (34.5) |
| **Private health insurance** |  |  |  |  |
| No | 140 | (22.8) | 145 | (23.6) |
| Yes | 475 | (77.2) | 469 | (76.4) |
| **Medical Maximizer-Minimizer Scale score** | 50 | (7.9) | 50 | (8.0) |

*Notes*: Frequency and column percentage (in parentheses) are presented for categorical variables, and means and standard deviations (in parentheses) are presented for continuous variables. SD, Standard deviations. No statistically significant differences were observed in any of the characteristics examined between the two groups at the 5% level.
